# Complementary feeding: a Global Network cluster randomized controlled trial

**DOI:** 10.1186/1471-2431-11-4

**Published:** 2011-01-13

**Authors:** Nancy F Krebs, K Michael Hambidge, Manolo Mazariegos, Jamie Westcott, Norman Goco, Linda L Wright, Marion Koso-Thomas, Antoinette Tshefu, Carl Bose, Omrana Pasha, Robert Goldenberg, Elwyn Chomba, Waldemar Carlo, Mark Kindem, Abhik Das, Ty Hartwell, Elizabeth McClure

**Affiliations:** 1 University of Colorado Denver, Aurora, CO, USA; 2 San Carlos University, Guatemala City, Guatemala; 3 RTI International, Research Triangle Park, NC, USA; 4 Eunice Kennedy Shriver National Institute of Child Health and Human Development, Rockville, MD, USA; 5 Kinshasa School of Public Health, Kinshasa, Democratic Republic of Congo; 6 University of North Carolina, Chapel Hill, NC, USA; 7 Aga Khan University, Karachi, Pakistan; 8 Drexel University College of Medicine, Philadelphia, PA, USA; 9 University Teaching Hospital, Lusaka, Zambia; 10 University of Alabama, Birmingham, AL, USA

## Abstract

**Background:**

Inadequate and inappropriate complementary feeding are major factors contributing to excess morbidity and mortality in young children in low resource settings. Animal source foods in particular are cited as essential to achieve micronutrient requirements. The efficacy of the recommendation for regular meat consumption, however, has not been systematically evaluated.

**Methods/Design:**

A cluster randomized efficacy trial was designed to test the hypothesis that 12 months of daily intake of beef added as a complementary food would result in greater linear growth velocity than a micronutrient fortified equi-caloric rice-soy cereal supplement. The study is being conducted in 4 sites of the Global Network for Women's and Children's Health Research located in Guatemala, Pakistan, Democratic Republic of the Congo (DRC) and Zambia in communities with toddler stunting rates of at least 20%. Five clusters per country were randomized to each of the food arms, with 30 infants in each cluster. The daily meat or cereal supplement was delivered to the home by community coordinators, starting when the infants were 6 months of age and continuing through 18 months. All participating mothers received nutrition education messages to enhance complementary feeding practices delivered by study coordinators and through posters at the local health center. Outcome measures, obtained at 6, 9, 12, and 18 months by a separate assessment team, included anthropometry; dietary variety and diversity scores; biomarkers of iron, zinc and Vitamin B_12 _status (18 months); neurocognitive development (12 and 18 months); and incidence of infectious morbidity throughout the trial. The trial was supervised by a trial steering committee, and an independent data monitoring committee provided oversight for the safety and conduct of the trial.

**Discussion:**

Findings from this trial will test the efficacy of daily intake of meat commencing at age 6 months and, if beneficial, will provide a strong rationale for global efforts to enhance local supplies of meat as a complementary food for young children.

**Trial registration:**

NCT01084109

## **Background**

### Research Justification

Among preventive measures that would reduce the excess mortality for children under the age of five years, exclusive breast feeding and good quality complementary feeding have been listed as first and third, respectively, with a calculated 600,000 deaths per year preventable by good complementary feeding (i.e. 6% of deaths) [1].

Infants and young children bear the brunt of chronic malnutrition and suffer the greatest consequences, that is, the highest risks of morbidity and mortality [2-4]. The incidence of malnutrition rises sharply between 6-18 months of age and the deficits acquired are difficult to compensate for later in the survivors [5]. In 2003, WHO/UNICEF published a Global Strategy for Infant and Young Child Feeding [6]. This document re-emphasizes that lack of exclusive breastfeeding in the first half of infancy is a major risk factor for infant/childhood morbidity and mortality, which is then compounded by inappropriate complementary feeding. It further indicates that inadequate knowledge about appropriate foods and feeding practices is often a greater determinant of malnutrition than actual lack of food. Emphasis is also given to the provision of sound and culture-specific nutrition counseling to mothers of young children in the widest possible use of indigenous foodstuffs that will help to ensure the optimal safe use of local affordable foods [7]. Recent significant, though still incomplete, progress with breastfeeding practices has not yet been matched in the area of complementary feeding.

Most intervention studies have not addressed local food-based strategies for the prevention of micronutrient deficiencies, although there have been successful education interventions focused on increased diversity that have shown promising results [8-10]. Yet millions of young children, especially those among the rural poor, do not have access to fortified foods or supplements and are unlikely to do so in the foreseeable future, especially on a sustainable basis. The WHO has published guidelines for complementary feeding which recommend daily intake of animal source foods after six months of age, noting that vegetarian diets cannot meet nutrient needs unless nutrient supplements or fortified products are used [11]. Even with diversity, expert reviews have concluded that it is not possible to achieve adequate intakes of the 'problem nutrients', specifically iron and zinc, with plant-based diets alone [12].

The importance of including animal source foods (ASF) in complementary feeding has been emphasized by several investigators [13-17]; by international organizations including WHO [18-20]; by national ministries of health as in Guatemala [21]; and by national committees including those in the U.S. [22-24]. Although these recommendations are inconsistent in details and lack specificity in regards to age of commencement, type of ASF, and quantities, they are in agreement on the value of the inclusion of ASF. Meat consumption has been positively associated with psychomotor outcome in children up to 24 months of age [25]; with iron status in late infancy [26]; and with improved growth and cognitive function in school-aged children [27,28]. As recently emphasized by The World Bank [29], however, it is at a younger age, i.e. the first one to two years when most damage results from malnutrition and when prevention is likely to be the most beneficial. The transition from exclusive breast feeding to a diversified diet, which is essential to satisfy nutritional needs, is a particularly vulnerable time [6,18].

Before addressing the challenges of an effectiveness study, which will depend on the identification of local sources of affordable meat and successful behavioral change communication, an efficacy trial to evaluate the theoretical benefits of meat as a first and regular complementary food from age six months is being undertaken in four diverse settings with high baseline stunting rates.

### Background information on the trial

The complementary feeding (CF) trial is being conducted as a common protocol through the Global Network (GN) for Women's and Children's Health Research. Centralized training was provided to country coordinators, who then provided local training to field staff. All sites followed a written manual of operations; identical equipment and supplies were provided to and utilized by all sites; common data forms were used and all data were transmitted to the data coordinating center (DCC) for compilation and storage.

### Trial sites

The trial is being implemented in Guatemala (Institute for Nutrition of Central America and Panama, Guatemala City), Pakistan (The Aga Khan University, Karachi), Democratic Republic of Congo (DRC, Kinshasa School of Public Health), and Zambia (University Teaching Hospital/University of Zambia School of Medicine, Lusaka). Community clusters were identified in rural areas in Zambia and DRC, small rural towns in the Western Highlands of Guatemala, and in urban/peri-urban communities in Pakistan. Stunting rates of at least 20% were defined *a priori *as the primary inclusion criterion for cluster selection. A second criterion for inclusion was the general lack of exposure of the community to micronutrient-fortified products (e.g. cereal grain products, infant formulas) at the time of recruitment.

### Pilot research

Prior to initiating the trial, a pilot study was conducted to obtain information about typical infant and toddler feeding practices in the potentially participating communities. Specifically, breastfeeding practices and use of meats and other animal source foods, micronutrient-fortified products, and vitamin-mineral supplements were documented for 5-9 month old infants and 12-24 month old toddlers. Anthropometric measurements were also obtained on the toddlers, with the aim of documenting stunting rates (Krebs NF, Mazariegos M, Tshefu A, Lokangaka A, Bose C, Sami N, Goldenberg R, Chomba E, Carlo W, Goco N *et al*: Intake of meat is associated with less stunting in toddlers in four diverse low income settings, submitted).

## Aim, objectives, and hypotheses

The overall objective of this study is to improve the quality of complementary feeding, and thereby improve growth and development of young children in low resource settings. The specific aim of this project is to determine the impact of a daily intake of meat between 6-18 months of age on linear growth velocity, dietary diversity, brain growth and neurocognitive development, infectious disease morbidity, and micronutrient status in populations traditionally dependent on non-micronutrient fortified plant-based foods for complementary feeding. The comparison group is infants from similar communities who receive an equi-caloric micronutrient fortified rice-soy food supplement.

The *primary hypothesis *is that daily feeding of meat from 6-18 months of age will result in significantly greater linear growth velocity compared to that achieved by daily feeding of an equi-caloric micronutrient fortified cereal supplement with both arms receiving education messages to optimize complementary feeding practices. *Secondary hypotheses *to be tested are that infants consuming meat daily will 1) have higher dietary diversity and variety scores; 2) higher scores on developmental testing; 3) lower rates of infectious disease morbidity; and 4) higher indices of micronutrient status for iron, zinc, and Vitamin B_12_.

## Methods/design

### Design

**Figure 1:**
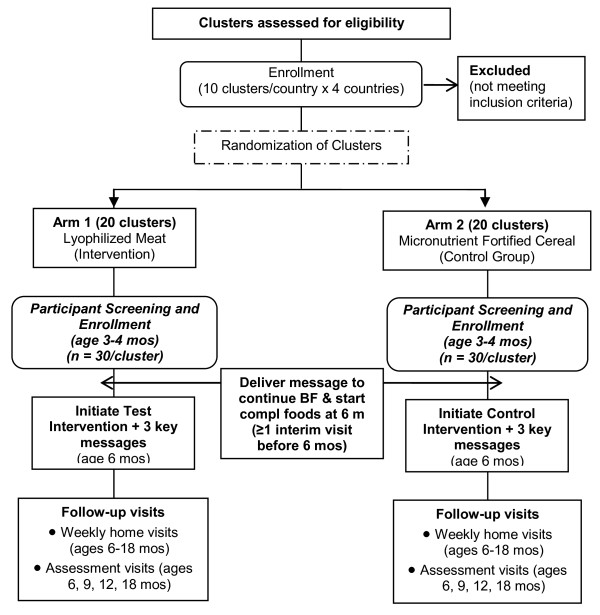
**Consort Diagram**.

The design is a non-masked cluster randomized controlled efficacy trial of a daily intake of lyophilized beef vs. an equi-caloric daily micronutrient fortified rice-soy based cereal from 6-18 months of age (Figure 1). Mothers and care providers of infants in both groups also receive three education messages during home visits by research workers and through illustrative posters for the home and posted at local health centers to reinforce selected features of WHO recommendations for complementary feeding [11]. An additional message to encourage exclusive breastfeeding until six months and to initiate complementary feeding at six months is given at the time of recruitment when the infants were three to four months of age.

The intervention is delivered by Community Coordinators, who are research workers with training as auxiliary nurses or equivalent primary community health training. The Community Coordinators are responsible for distribution of the complementary foods to the participating mother and infant pairs in their corresponding community/cluster; teaching the mothers how to prepare the food; communicating the educational messages during home visits; collection of baseline data; monitoring participant compliance with intervention food; and collecting weekly infectious disease morbidity data.

Trained staff different from the intervention teams conduct standardized assessments of infants at 6, 9, 12, and 18 months of age. Assessments include anthropometric measurements; Infant and Child Feeding Index (ICFI) and its components: Food Variety Score (FVS) and Dietary Diversity Score (DDS) [30,31]; measures of neurocognitive development at 12 and 18 months; and biochemical indices of micronutrient status at 18 months.

### Cluster Randomization and Sampling

The DCC randomized participating clusters within each country to either the lyophilized meat or cereal intervention using toddler stunting rates as the matching criterion; all clusters had stunting rates greater than 20% (Figure 1). Records of all births within each cluster were obtained from the Global Network Birth Registry or through local health center records by senior team members starting as soon as cluster randomization was completed and continuing as long as necessary to enroll the goal number of infants. Each month, a list of potential participants was randomly selected from these records for screening and recruitment.

### Intervention

#### Test group: Meat Supplement

A cooked, diced, lyophilized beef product is provided for daily consumption. It was expected that the infant would consume the initial target quantity of 15 g lyophilized beef/day by 7 months of age. Fifteen grams of the lyophilized beef is equivalent to 30 grams of cooked meat. The daily portion of lyophilized meat is increased to 22.5 g per day at 12 months of age. The lyophilized product is easily crumbled into a powder, which, when moistened, provides a paste or puree consistency that is readily consumed by a young infant. For older infants and toddlers, the cubes are mixed in with other foods or eaten dry as a "finger food." Mothers and care providers are taught age appropriate preparations of the product by the community coordinators during home visits. In the early weeks of the study, the meat was offered to the infant as the only complementary food or with a minimum of other foods, in order to achieve the intake goal of 15 g per day. The nutrient content of the lyophilized beef is provided in the Table 1.

**Table 1:** Nutrient composition per daily serving of rice-soy cereal and lyophilized beef

Nutrient	6 - 12 mos of age			13 - 18 mos of age		
	Cereal*	**Beef**	**RNI per day**	Cereal*	**Beef**	**RNI per day**

Serving Size, g, uncooked	20	15	NA	30	22.5	NA

*Macronutrient Composition*						

Energy, Kcal	70	70	NA	105	105	NA
Protein, g	3.07	13	NA	4.60	19.5	NA
Fat, g	0.13	1.7	NA	0.19	2.5	NA
Carbohydrate, g	13.92	0	NA	20.89	0	NA

*Minerals*						

Zinc, mg	2.2	2.0	2.5 - 4.1	3.3	3	2.4 - 4.1
Iron, mg	5.5	1.4	6.2	8.3	2.1	3.9
Copper, mg	0.12	0.02		0.18	0.03	
Selenium, μg	4	NA	10	6	NA	17
Magnesium, mg	20	4.5	54	20	0.7	60

*Vitamins*						

Riboflavin, mg	0.07	0.06	0.4	0.11	0.09	0.5
Thiamine, mg	0.07	0.02	0.3	0.11	0.03	0.5
Niacin, NE	1.2	0.6	4	1.8	0.9	6
Vitamin B6, mg	0.09	0.06	0.3	0.5	0.09	0.5
B12, μg	0.10	0.56	0.5	0.15	0.84	0.9
Vitamin C, mg	70	0	30	105	0	30
Pantothenate, mg	0.14	0.08	1.8	0.21	0.12	2.0
Biotin, μg	0.58	N/A	6	0.87	NA	8
Folate, DFE, μg	17	1.3	80	26	2.0	160
Vitamin E (α-TE), mg	2	NA	No RNIs	3	NA	No RNIs

*Source: All micronutrient fortification levels in cereal are based on recommendations by Lutter and Dewey [32] for 6-23 mo olds.

The lyophilized meat used at all sites in this study is commercially prepared and marketed by Mountain House, Inc (Portland, Oregon, USA). The contents are stable for 15-20 years if the can is unopened and stored in temperatures less than 100°F. Once opened, the product is useable for up to 10 days when stored at room temperature. The manufacturer obtains meat only from US sources. A United States Department of Agriculture (USDA) inspector is on premises at the plant at all times to certify the quality and safety of the product, resulting in an USDA seal of certification on all cans. An international halal certification was obtained from the Islamic Services of America for the meat exported to Pakistan for use in the study. This certification signifies the compliance with the most stringent guidelines for the animal sacrifice, and cooking and packaging of the meat product.

The lyophilized beef was shipped from the U.S. "door to door" in a single lot to each participating site. Sites identified a secure, temperate (< 100°F) facility in which to store the cans of meat. Each site was responsible for obtaining import permits specific for their country.

From the 476 gram cans of lyophilized meat one member of the research staff weighs out daily portions of lyophilized meat into small zip-lock plastic bags (appropriately labeled and provided by study).

#### Comparison Group: Micronutrient Fortified Rice-Soy Cereal Supplement

The cereal supplement portion is iso-caloric to the meat supplement and provides approximately 70 kcal/day in a 20 g (uncooked) portion size, which is increased to 30 g/day when the infant reaches 12 months of age to match the energy provided by the increased portion of meat. The product, which was formulated specifically for this study by Nutrica, Inc (Guatemala City, Guatemala), is a mixture of pre-cooked rice and soy flour, fortified by the manufacturer according to recent guidelines for multiple micronutrients in complementary foods for 6-23 month olds [32]. Nutrient composition of 20 g and 30 g portions of the rice-soy mix are given in Table 1. The zinc and iron concentrations of the cereal mix were verified by laboratory analysis at the University of Colorado Denver.

According to the manufacturer preparation specifications, the 20 g serving, provided as a single-serving packet, is dissolved in ~120 ml clean water, brought to a boil and allowed to cook over low heat for 1-2 minutes prior to consumption. Pilot studies of preparation and acceptability in each site demonstrated good acceptance.

To increase sanitation and protection from pests, participants in both intervention arms are provided a tightly sealed container in which to store the weekly supply of food packets. A small metal cooking and serving pan (Sierra Cup™, Coghlan's Ltd.,

Winnipeg, MB, Canada) and a plastic infant spoon are also provided for infants in both groups; these both fit in the container along with the study food. Pictorial instructions for food preparation (specific to the assigned study group) and hand-washing (mother and infant) are affixed to the sealable container.

The week's supply (7 portions) of the meat or cereal is delivered to homes by community coordinators or their assistants. Intervention food preparation and consumption by the infant are observed during home visits by the research staff on a daily basis for the first two weeks of the intervention; 3 times per week for the first 3 months; and then weekly for the remainder of the intervention. During these home observations, intake of study food is recorded. If the infant does not consume the entire daily allotment at the observed feed, the mother is asked to safely store any remaining meat and to offer it to the same child later in the day.

Compliance is monitored for both study foods by counting and recording the number of unused packets (zip-lock or cereal packages) at the time of delivery of the next week's supply.

### Dissemination of Three Selected Education Messages

Both treatment groups receive three educational messages to encourage proper infant and child feeding. The educational messages were adapted from the *Guiding Principles for Complementary Feeding *[11] and other WHO educational materials [18]. The three messages are to: 1) feed thickened gruels every day; 2) feed the infant/toddler complementary foods (in addition to breastfeeding) at least 3 times a day; and 3) choose a variety of local foods for the infant/toddler. For both arms and all communities, posters were developed specifically for this study. The words were translated into the local language, and pictures were tailored to the local culture. Small posters are provided to all participating families, and identical larger posters are placed in local health centers to reinforce the messages and principles. Community health workers introduce these concepts one at a time as progressively appropriate for the infant's developmental stage, and as the mothers mastered each skill.

### Staff Training

After an initial introduction to the goals and objectives of the study research teams at a Global Network Steering Committee meeting, representatives of each of the participating sites traveled to the University of Colorado Denver for comprehensive training. Training modules included research ethics; procedures for recruitment, informed consent and enrollment of mothers and infants; instructions for storage, dispensing, and preparation of study foods; administration of data forms, including the administration and scoring of the Infant Child Feeding Index; and training relating the three education messages to infant feeding development and the principles of responsive feeding. and monitoring compliance of study procedures. Training and certification in anthropometric measurements and use of the equipment provided by the study were conducted. Staff was also familiarized with the developmental tests, as described in more detail below. Procedures for venipuncture and handling blood samples, including precautions to avoid zinc contamination of samples, were also demonstrated and written instructions were provided.

The Global Network Senior Foreign Investigators and country coordinators were provided the materials required to replicate training of community coordinators and other staff at their sites. Training was specific for the intervention teams and for the assessment teams in order to focus on the necessarily different skills required for data collection. The lead Global Network team (Colorado-Guatemala) and RTI provided technical assistance throughout the training to ensure that training was properly conducted and procedures were standardized.

### Study Subjects and Enrollment

When infants were 3 months of age, caregivers were approached by community coordinators, in the order on the list of randomly selected potential participants generated by the DCC, to recruit for participation in this study. Thirty infants per cluster provided informed consent and were enrolled over a period of approximately 10 months.

Inclusion criteria for individual families were to have an infant aged 3-4 months who was exclusively or predominantly breastfed and with the mother's intent to continue breast feeding through at least one year. Exclusion criteria were any family receiving or likely to receive free or subsidized complementary foods (or infant formula) through the health system or non-governmental organizations, and those who were feeding or intended to feed fortified infant formula or micronutrient-fortified commercial complementary foods. Exclusion criteria for individual infants were congenital anomaly; infant of multiple births; or neurological deficit apparent at the time of enrollment.

This research study was reviewed for approval by the Institutional Review Board (IRB) of each participating institution. All participants gave informed consent prior to enrollment into the study.

### Duration

At least one interim home visit was conducted between enrollment and the start of the intervention when the infant reached 6 months. The first formal assessments (anthropometry and dietary data collection) were obtained at 6 months of age. At this time, the complementary feeding intervention for both groups was initiated and continues through 18 months of age. Follow-up anthropometric assessments are conducted at 9, 12, and 18 months of age. Neurocognitive assessments are performed at 12 and 18 months. Blood samples to assess nutritional status are being obtained from a convenience sample at completion of the trial at 18 months of age.

### Power Analyses

Based on the effect size of 0.5 standard deviation (SD) units observed in another cluster based study of complementary feeding [8], the statistical power for this study was set at 80% to detect a more conservative difference of 0.3 SD in linear growth velocity from 6 to 18 months of age between the intervention and control groups, using a two-tailed test and a 5% Type I error rate.

An ICC = 0.05 was assumed for the sample size calculation. Note that 0.05 is in the upper ranges of ICC values observed in many cluster randomized trials, and would provide the most conservative (i.e., the largest) sample size estimate. Moreover, for a given level of ICC (and Type I and II errors) in a cluster randomized study, increasing the average cluster size reduces the number of required clusters, while increasing the total sample size. This fact, coupled with the consideration that a minimum of 10 clusters are required per treatment group to credibly estimate the ICC with acceptable precision, plus the actual cluster sizes observed in ongoing GN trials, led to an estimated average cluster size of 30.

The above assumptions, an adjustment for 20% attrition over the duration of the study, plus an allowance for cluster loss because of unforeseen reasons such as natural disasters (2 per treatment group), result in a requirement of 20 clusters per treatment group, for a total of approximately 600 subjects per treatment group (40 clusters and 1200 subjects total). Thus, 10 clusters (5 per intervention arm) in each participating country, with approximately 30 subjects per cluster were necessary to achieve desired power.

### Outcome Assessments to Test Hypotheses

Evaluations are undertaken by two teams per site of specially trained community research workers who were recruited from the participating communities. Clusters are randomly assigned to each assessment team, so that each team conducts data collection on both meat and cereal clusters.

The assessment teams are responsible for data collection and measurements for all study participants. The assessment teams do not participate in implementation of the study interventions. Participants' caregivers are asked to come to a central site (e.g. community health center facilities) for assessments.

#### Anthropometry

All measurements are made according to standardized World Health Organization (WHO) guidelines [33]. Anthropometric measurements include naked weight, recumbent length, and head circumference. Naked weights are recorded using a Seca 334 infant scale accurate to 5 grams (Perspective Enterprises, Portage, MI). Recumbent length is measured with a Seca infantometer (model 416, accurate to 1 mm, Perspective Enterprises, Portage, MI). Head circumferences are measured with a plasticized non-elastic measuring tape (accurate to 1 mm) to the nearest 0.1 cm. Each site was provided with the anthropometry equipment. Duplicate measurements are made for each growth parameter. If the two initial measurements differ by more than the pre-determined allowance (0.4 cm, 10 g, and 0.2 cm for length, weight and head circumference, respectively), a third measurement is undertaken. Anthropometric instruments are calibrated regularly.

#### Dietary Data and Infant and Child Feeding Index (ICFI)

Qualitative 24-hour dietary recalls for the previous day [30,31] are obtained at 6, 9, 12 and 18 months. Dietary data collected for the ICFI are: breast feeding (yes/no, number of times per day); bottle/cup feeding (yes/no, number of times per day); numbers of complementary feeds; composition of meals/snacks. This qualitative 24-hr recall is simple to obtain without the assistance of a research nutritionist. From these data, a food variety score and dietary diversity score are calculated. Consumption of micronutrient-fortified products and the administration of supplements are also monitored. The methods used for generating the feeding index scores will be those of Ruel and Menon [31], which have been applied to a large-scale study in Guatemala [34] and have been adapted for use in Sub-Saharan Africa and Pakistan [30].

#### Ages and Stages Questionnaire

The Ages and Stages Questionnaire (ASQ) and ASQ Social-Emotional (ASQ-SE) are verbally administered to all mothers to compare infant neurodevelopment between the two intervention arms at 12 and 18 months. The ASQ, 2^nd ^ed. [35], is a 30-item instrument addressing parent-reported child development in the domains of communication, gross motor, fine motor, problem solving, and personal-social development using age-specific forms. The ASQ-SE scale uses 22-30 items (depending on age) to assess parent-reported child social and emotional functioning in the areas of self-regulation, compliance, communication, adaptive functioning, autonomy, affect, and interaction with people. The ASQ and ASQ-SE assess children in their natural environments to ensure valid results. The questionnaire is available in English, Spanish, and French, and was translated to local dialects for the sites as necessary. A major advantage to the ASQ is its simplicity for administration, which does not require a trained professional, and it has also been used in studies in developing countries [36,37]. Minor modifications have been made to the questions to adapt to local settings. All modifications were reviewed by an external expert in child development who is a consultant to the Global Network.

#### Bayley Scales of Infant Development

The Bayley Scales of Infant Development, 2nd Edition, (BSID II) are administered to assess infant development at 18 (± 0.5) months of age. All sites have obtained a standardized BSID II test kit, instructions and evaluation/scoring forms. The test administration is conducted at each site in a consistent location adapted and arranged to provide a standardized environment for the testing. These are performed by research team members with standardized professional training, to minimize variability within and among clusters. The individuals administering the BSID II at all sites have undergone training by an appropriately trained and experienced professional (e.g. psychologist). Three of the participating sites have prior experience with the BSID II, and past experience within the Global Network has been utilized to conduct standardized training, quality control and assurance of validation and reliability of testing procedures. Video recordings of the administration of the BSID II have been made of each trainee, and these recordings have been reviewed by the child development consultant. For a given site, the same individuals administer the BSID to all participants. To avoid inter-examiner bias, assessment teams have been randomly assigned to complete evaluation visits for meat and cereal clusters. The evaluators administer the BSID directly to each child in the appropriate language using standard material prescribed in the manual, with minimal adaptations made to local settings.

#### Biomarkers of Micronutrient and Inflammation Status

Biomarkers to assess nutritional and inflammatory status include hemoglobin, serum ferritin and transferrin receptor for iron status; serum zinc; serum Vitamin B_12_; and C-reactive protein. Three mL of blood are collected by antecubital venipuncture approximately two hours after eating from a convenience sample of participants (80 from meat and 80 from cereal arms for each country) at 18 months of age (± 2 weeks). Hemoglobin levels are measured immediately utilizing a HemoCue system (HemoCue, Inc., Lake Forest, CA). Blood draws are not obtained from any obviously ill child and, if necessary, are rescheduled within the timeframe above. All laboratory analyses are being performed in Colorado in the Pediatric Nutrition Laboratory (University of Colorado Denver). Standardized procedures have been implemented at each site to assure cold chain integrity between collection and receipt in Colorado.

#### Infectious Disease Morbidity

Data on incidence and duration of infant diarrhea (more than 3 watery stools per day) are collected weekly from 6 to 18 months based on mother's recall. The mothers are also asked several questions regarding presence of respiratory symptoms (cough, tachypnea/dyspnea, lower chest wall in-drawing and fever) to assess for lower respiratory infection, and regarding symptoms suggestive of malaria. These data are collected in the home by the community coordinators.

#### Process evaluation

Exit interviews are conducted at each site after the 18 month visit for a randomly selected subset of participating mothers. The interviews evaluate the mothers' perceptions about the intervention, the education messages, and the overall experience of study participation. External events that could potentially impact the study outcomes are monitored and documented in quarterly reports by the field coordinators and senior investigators at each site.

### Data management

Data forms are completed using pen and paper. Completed data forms are collected and reviewed weekly by the field supervisor at each research site. Reviewed forms are keyed into a data management system developed by the DCC (RTI). Research sites store data in a stable and secure server, with redundant power supplies, dual network cards, redundant hard drives, and a semi-automated backup procedure to backup server data on a daily basis. Data are transferred from the field sites to the DCC at least once a week using an automated file transfer system. All database files are password protected, compressed, encrypted and signed with a unique digital certificate using public key cryptography before transmission, providing strong protection during transmission.

The DMS includes a variety of data validation, including range checks, data type checks and customized skip patterns. Multiple languages (e.g., English, Spanish, and French) are supported, allowing local data entry personnel to view and enter data in their native language. Data validation is performed both as data are entered and when each form is saved. Validation errors are recorded in a data table for later analysis. A running journal of all data entry is kept, allowing the system to be rolled-back to any given point in time.

The DMS includes reports to assist with project management that can be generated by the research sites. These include reports on complete and incomplete forms, missing forms, scheduled appointments, and errors. Like other parts of the system, all reports are available in the native language.

The DCC converts the databases from Microsoft Access into SAS for analysis. For each database, a SAS program is written which converts the Access database to SAS files, creates date and time variables, and assigns labels and formats to each variable. The SAS files are then stored on the project share in the appropriate protocol directory. These programs are run automatically each time RTI receives a data transmission.

### Data analyses

All analyses will be conducted using the intent to treat approach. The primary analysis will be adjusted only for clustering (the community effect). This adjustment for clustering also adjusts for community or site differences. A comprehensive analysis of baseline variables of the two treatment groups will be performed to assess the extent, if any, of baseline imbalances between the two groups of clusters/communities assigned to the two treatment groups, both at the individual and at the cluster levels, to determine the need for adjusting analyses. The baseline variables to be examined include: gender and socioeconomic status (SES), prevalence of exclusive breast feeding at 6 months, birth weight, maternal stature, parity, and site. Imbalances occurring at the cluster level can also be adjusted for by incorporating cluster level covariates, which may be cluster aggregated versions of the co-variables mentioned above.

For analysis of the primary outcome, the WHO Multicentre Growth Reference Study [38] will be used as a comparison. Mean Z-scores at 6, 9, 12 and 18 months for length-for-age, weight-for-age and weight-for-length will be determined as well as changes in Z-scores over 12 months. Linear growth velocity from 6-18 months of age was selected as the primary outcome measure because of its potential to be statistically more powerful than a cross-sectional comparison of length at 18 months alone.

The statistical analyses of the primary outcome and other comparison between treatment groups will use hierarchical or multi-level models to adjust for the cluster randomized design of the study. Hierarchical modeling provides a straightforward framework to accommodate adjustments for potential covariates and confounders, both at the subject level (e.g., birth weight, gestational age, gender, etc.) and at the cluster/community level (average SES at the community level, community access to prenatal and antenatal care, community exposure to other nutrition messages, etc.). For both 18-month and longitudinal outcomes, linear mixed models will be used for continuous outcomes such as growth parameters, whereas generalized linear mixed models will be used for discrete outcomes such as frequency of various nutritional intakes and incidence of morbidities.

## Discussion

### Administrative Structures

#### Trial Steering Committee

A Complementary Feeding Steering Committee provided guidance for the development of the study protocol and study procedures in the preparatory phase of the trial, and provides ongoing scientific oversight. The committee meets in-person two times per year to discuss study progress, problem-solve, and provide guidance where necessary.

#### Data Monitoring Committee

As per the NIH Policy for Data Safety and Monitoring, the trial is overseen by a data monitoring committee (DMC). The DMC has the responsibility to ensure the safety of study participants and the validity and integrity of data collected. The DMC determines the safe and effective conduct and recommends conclusion of a trial when significant benefits or risks have developed or the trial is unlikely to be concluded successfully.

#### Research ethics

This research study was reviewed for approval by the Institutional Review Board (IRB) of each participating institution. Annual reviews are conducted as per each institution's standard review procedures. The IRBs are registered at OHRP with FWAs. Safety and efficacy analyses are performed at scheduled intervals based upon age (after every 100 infants reached one year of age) and the completion of study participation. All participants gave informed consent prior to enrollment into the study.

#### Implications

A strong evidence base will be required to mobilize action at local, national and international levels to develop local meat production at a household or community level. The lyophilized beef greatly simplified the implementation of this efficacy study, but it is recognized that the provision of this product is not sustainable. However, positive results from this study will provide an impetus to advocate for development of locally sustainable means and incentives to modify current practices so that the older infant and toddler, the primary beneficiaries, are provided some form of meat as a complementary food.

Trial registration: NCT01084109

## Abbreviations

ASF, Animal source foods; ASQ, Ages and Stages Questionnaire; ASQ-SE, Ages and Stages Questionnaire - Social Emotional; BSID, Bayley Scale of Infant Development; CF, Complementary feeding; DCC, Data coordinating center; DMC, Data monitoring committee; DMS, Data monitoring system; FWA, Federal Wide Assurance; GN, Global Network; ICC, Intra-cluster correlation; ICFI, Infant and Child Feeding Questionnaire; IRB, Institutional Review Board; OHRP, Office for Human Research Protections; SES, Socio-economic status; USDA, United States Department of Agriculture; WHO, World Health Organization.

## Competing interests

The authors declare that they have no competing interests.

## Authors' contributions

NFK, KMH, MM, and JEW conceived of and designed the study, obtained funding and designed the intervention and evaluation procedures. LLW, MKT, AT, CB, OP, RG, EC, and WC participated in the design of the study, the intervention and evaluation procedures, and in the study implementation. AD, HC, NG, TDH, EM, and MK participated in the study design, data management and statistical analysis. The remaining members of the Complementary Feeding Study Group participated in the design and implementation of the study. This protocol was reviewed and approved by NIH as part of the grant application process. Additionally, NIH had input into the final design of the study protocol prior to implementation. All authors have read and approved the manuscript.

## Pre-publication history

The pre-publication history for this paper can be accessed here:

http://www.biomedcentral.com/1471-2431/11/4/prepub
